# Identification and characterization of mRNAs and lncRNAs in the uterus of polytocous and monotocous Small Tail Han sheep (*Ovis aries*)

**DOI:** 10.7717/peerj.6938

**Published:** 2019-05-23

**Authors:** Yongfu La, Jishun Tang, Xiaoyun He, Ran Di, Xiangyu Wang, Qiuyue Liu, Liping Zhang, Xiaosheng Zhang, Jinlong Zhang, Wenping Hu, Mingxing Chu

**Affiliations:** 1 Chinese Academy of Agricultural Sciences, Key Laboratory of Animal Genetics and Breeding and Reproduction of Ministry of Agriculture, Institute of Animal Science, Beijing, China; 2 Gansu Agricultural University, College of Animal Science and Technology, Lanzhou, China; 3 Anhui Academy of Agricultural Sciences, Institute of Animal Husbandry and Veterinary Medicine, Hefei, China; 4 Tianjin Institute of Animal Sciences, Tianjin, China

**Keywords:** LncRNAs, Prolificacy, RNA-Seq, Small Tail Han sheep, Uterus

## Abstract

**Background:**

Long non-coding RNAs (lncRNAs) regulate endometrial secretion and uterine volume. However, there is little research on the role of lncRNAs in the uterus of Small Tail Han sheep (*FecB*++). Herein, RNA-seq was used to comparatively analyze gene expression profiles of uterine tissue between polytocous and monotocous sheep (*FecB*++) in follicular and luteal phases.

**Methods:**

To identify lncRNA and mRNA expressed in the uterus, the expression of lncRNA and mRNA in the uterus of Small Tail Han sheep (*FecB*++) from the polytocous group (*n* = 6) and the monotocous group (*n* = 6) using RNA-sequencing and real-time polymerase chain reaction (RT-PCR). Identification of differentially expressed lncRNAs and mRNAs were performed between the two groups and two phases . Gene ontology (GO) and pathway enrichment analyses were performed to analyze the biological functions and pathways for the differentially expressed mRNAs. LncRNA-mRNA co-expression network was constructed to further analyses the function of related genes.

**Results:**

In the follicular phase, 473 lncRNAs and 166 mRNAs were differentially expressed in polytocous and monotocous sheep; in the luteal phase, 967 lncRNAs and 505 mRNAs were differentially expressed in polytocous and monotocous sheep. GO and KEGG enrichment analysis showed that the differentially expressed lncRNAs and their target genes are mainly involved in ovarian steroidogenesis, retinol metabolism, the oxytocin signaling pathway, steroid hormone biosynthesis, and the Foxo signaling pathway. Key lncRNAs may regulate reproduction by regulating genes involved in these signaling pathways and biological processes. Specifically, *UGT1A1*, *LHB*, *TGFB1*, *TAB1*, and *RHOA*, which are targeted by *MSTRG*.*134747*, *MSTRG.82376*, *MSTRG.134749*, *MSTRG.134751*, and *MSTRG.134746*, may play key regulatory roles. These results offer insight into molecular mechanisms underlying sheep prolificacy.

## Introduction

Small Tail Han sheep is an excellent local breed in China, with high fecundity and year-round estrus. The average litter size of Small Tail Han sheep is 2.61, and the average lambing rate is 286.5% (Di et al., 2012). Most Small Tail Han sheep are polytocous, although a few are monotocous. To date, mutations in *BMP15*, *GDF9* and *BMPR-1B* have been found in some sheep breeds as genes affecting fecundity. *FecB* is a key candidate gene for the genetic control of sheep reproductive performance, and is known as the major gene associated with sheep prolificacy (Mulsant et al., 2001; Wilson et al., 2001). Recent studies have shown that *FecB* gene has a close relationship with the litter size of Small Tail Han sheep (Guo et al., 2018). Therefore, based on the FecB genotyping in our study, we selected Small Tail Han sheep with different fecundity as experimental materials.

Development of high-throughput transcriptome analysis over the past few years has resulted in lncRNAs receiving extensive attention because they are a novel regulator of cell development (Veneziano, Nigita & Ferro, 2015). Long non-coding RNAs (lncRNAs) are greater than 200-nt long and have essential regulatory functions. In animals, lncRNA expression is lower than that of normal coding genes but has more functions than that was previously recognized. Some studies found that lncRNAs played essential roles in sheep reproduction, and many lncRNAs from ovarian tissue or germ cells have been identified in sheep (Feng et al., 2018; Miao et al., 2016a; Miao et al., 2016b). Yang et al. (2018) identified 1118 lncRNAs and 7253 mRNAs in the testes of sheep with premature and mature regulators of testis development and spermatogenesis. Feng et al. (2018) identified five lncRNAs and 76 mRNAs in the ovarian tissues of Hu sheep with high and low prolificacy, respectively. Miao et al. (2017) analyzed the lncRNA and mRNA expression profiles in the ovaries of Dorset ewes (low fecundity) and Small Tail Han ewes (high fecundity) with genotypes BB and ++. lncRNAs are abundant in the uterus, and the total number of developmental changes are similar to those in mRNAs (Wang et al., 2017). Identification and functional analysis of lncRNAs and mRNAs have been conducted in uterine tissue of humans (Zhou et al., 2016), mice (Wang et al., 2017), and pigs (Wang et al., 2016). However, there are few studies on the lncRNA in the uterus of sheep. Recently, in several studies, lncRNAs target genes and mRNAs were shown to be significantly enriched in the ovarian steroidogenic pathway (Gareis et al., 2018), retinol metabolism (Huang et al., 2003), oxytocin signaling pathway (Miao et al., 2016a; Miao et al., 2016b), and steroid hormone biosynthesis (Chen et al., 2015), which are associated with the uterus and ovarian function. Those studies revealed that lncRNAs might regulate ovarian and uterine function, and therefore regulate livestock reproduction.

In this study, RNA-Seq was used to comparatively analyze the gene expression profiles of uterine tissue between high and low fecundity Small Tail Han sheep (*FecB* ++). By conducting Gene Ontology (GO) and Kyoto Encyclopedia of Genes and Genomes (KEGG) pathway enrichment analyses, and co-expression network analysis, we studied the molecular mechanisms of differentially expressed lncRNAs and genes in uterine tissue that affect prolificacy. These results could provide useful information for studying the relationship between lncRNA regulation and prolificacy in sheep.

## Materials & Methods

### Samples

All experiments were performed following the relevant guidelines and regulations set by the Ministry of Agriculture of the People’s Republic of China (No. IASCAAS-AE-03).

Based on the TaqMan assay using the *FecB* mutation probe, a total of 12 pluriparous ewes with *FecB* ++ genotypes were selected from nucleus herds of Small Tail Han sheep in the southwest region of Shandong Province, China. These ewes were all approximately three years old and weighed 63 kg. Ewes were divided into two groups: a polytocous group (PG, *n* = 6, litter size and ovulation number ≥ 2) and a monotocous group (MG, *n* = 6, litter size and ovulation number = 1) based on ovulation number and three lambing records. All animals had free access to water and food under natural lighting.

All ewes were treated with synchronous estrus. A vaginal sponge was first implanted for 12 days. Estrus was then tested by a ram each day. These ewes were then divided into follicular and luteal phase groups. Uteri from six ewes (three polytocous ewes and three monotocous ewes) were collected between 45 and 48 h (follicular phase; PF and MF, respectively). Additionally, uteri from the other six ewes (three polytocous ewes and three monotocous ewes) were collected on a ninth day (luteal phase; PL and ML, respectively). All samples were immediately stored at −80 °C for total RNA extraction.

### RNA extraction, library construction, and RNA-seq

Total RNA was extracted from the uterus of 12 ewes using TRIzol (Invitrogen, Carlsbad, CA, USA) according to the manufacturer’s instruction. RNA concentration was measured using the Kaiao K5500 spectrophotometer (Beijing Kaiao Technology Development Co., Ltd, Beijing, China). RNA integrity was assessed using the RNA Nano 6000 Assay Kit of the Agilent Bioanalyzer 2100 System (Agilent Technologies, Santa Clara, CA, USA).

The rRNA was depleted from 3 µg of total RNA using Ribo-Zero™ Gold Kits (Epicentre, Madison, WI, USA). Sequencing libraries of the 12 samples (PF, *n* = 3; MF, *n* = 3; PL, *n* = 3; ML, *n* = 3) were generated using NEB Next Ultra Directional RNA LibraryPrep Kit for Illumina (NEB, Ipswich, MA, USA) according to the manufacturer’s instructions, and index codes were used to label the sequences of each sample. After cluster generation, the library preparations were sequenced on an Illumina Hiseq platform (Illumina, San Diego, CA, USA). Raw data of the performed RNA-seq have been recorded in the SRA public database (Accession number:  SRP173986).

### Reference genome mapping and transcriptome assembly

Raw data in fastq format were processed through in-hoseperl scripts. In this step, clean reads were obtained by removing reads with adapter contamination, reads that contained poly-N, and low-quality reads from raw data. Simultaneously, the Q20, Q30, and GC contents of the clean data were calculated. All downstream analysis was based on high-quality clean data. HiSAT2 (Pertea et al., 2016) was used to align clean reads of each sample to the sheep reference genome *Oar_v3.1*. StringTie (Pertea et al., 2015) was used for transcriptome assembly and reconstruction. Thus, known lncRNA and mRNA transcripts were identified, and the position of transcripts was obtained.

### Identification of potential lncRNA candidates

LncRNAs were identified using the following workflow. (1) Transcripts >200-nt long with >2 exons were obtained. (2) Transcripts with a coverage less than 5 in all samples were removed. (3) The different classes of class_code annotated by “u”, “i”, and “x” were retained, which corresponded to lincRNAs, intronic lncRNAs, and anti-sense lncRNAs, respectively. (4) Used Gffcompare to compare with annotation files to screen out known mRNAs and other non-coding RNAs (e.g., rRNAs, tRNAs, snoRNAs, snRNAs). Transcripts without coding potential, as predicted by CNCI, CPC, PFAM, and CPAT, were candidate lncRNAs.

### Differentially expressed gene analysis

The FPKM was used to normalize the expression levels of lncRNAs and mRNAs, which eliminated the effect of sequencing depth, gene length, and sample difference on gene expression levels (Trapnell et al., 2010). For experiments with three biological replicates, the differentially expressed lncRNAs and mRNAs were identified using the R package DEseq (Anders & Huber , 2010) after the negative binomial distribution. For biological replicates, lncRNAs and mRNAs with *P* <0.05 and Fold change >1.5 were considered differentially expressed between the polytocous and monotocous ewes of different estrus cycles.

### GO and KEGG pathway enrichment analysis of differentially expressed genes

Gene Ontology (GO) enrichment analysis of differentially expressed genes or lncRNA target genes was implemented by the GOseq R package, in which gene length bias was corrected (Young et al., 2010). GO classifies functions into three groups: cellular components, molecular functions, and biological processes. The KEGG biological pathways database (http://www.genome.jp) is a central public database for understanding high-level functions and regulatory network research. Enrichment analysis was performed on each Pathway in KEGG using a hypergeometric test. The calculated *P* value and 0.05 being defined as the significant threshold, the genes were screened and enriched for the pathways.Next, the significance of the pathway enrichment analysis was corrected by FDR, and the corrected *P*-value (*Q*-value) was obtained. Differentially expressed genes were further studied using the GO and KEGG databases to study the functions of the genes and identify the pathways in which they participate. If a *P* value was ≤0.05, enrichment was considered significant.

### Prediction and functional analysis of differentially expressed lncRNA target genes

The primary role of lncRNAs, which are a type of noncoding RNA, is to regulate their target genes by cis-regulating nearby protein-coding genes and trans-regulating distal protein-coding genes. Protein-coding genes located 50-kb upstream and downstream of a lncRNA in a genome are cis-target genes, whereas protein-coding genes with a correlation coefficient >0.9 with a lncRNA were trans-target genes (Fatica & Bozzoni, 2013). GO annotation and KEGG pathway enrichment analyses were performed on the obtained lncRNA target genes to identify the biological processes and signaling pathways enriched in the lncRNAs. Then, we predicted the functions of lncRNAs.

### LncRNA–mRNA network construction

To further explore the interactions between the lncRNAs, target genes and differentially expressed genes in sheep reproduction. Based on the targeting relationship between mRNA and lncRNA, we screened networks related to uterine function and reproduction with reference to their GO and KEGG enrichment terms and keywords for classification. Visualization of gene interactions is achieved through an open software platform called Cytoscape (V3.1.1) (Saito et al., 2012).

### Gene expression validation by quantitative real-time PCR

We used qRT-PCR to verify gene expression levels. We used approximately 0.1 µg of each RNA sample and reverse transcribed into cDNA using RT reagent. Real-time PCR was performed at 95 °C for 10 min, followed by 95 °C for 15 s, 60 °C for 60 s for 45 cycles, and 72 °C for 30 s. qPCR was performed on the LightCycler 480II (Roche, Basel, Sweden) using SYBR Green Real-time PCR Master Mix (TOYOBOCO, LTD, Osaka, Japan). β-Actin was used as an internal reference to normalize target gene expression. All experiments were performed in triplicate. mRNA and lncRNA primers are shown in Table 1.

**Table 1:** Details of primer sequences and expected product sizes of genes used for qRT-PCR.

Genes and lncRNAs	Primer sequence (5′–3′)	Product size (bp)
CYP1A1	F: CCTGGAGACCTTCCGACACT R: ATCTGCCACTGGTTCACAAA	126
PTGS2	F: CCCAGCACTTCACCCATCAA R: CAGACCAGGCACCAGACCAA	290
CDC20	F: GCAGACCTTCACCCAGCATC R: GCATCCACGGCACTCAGACA	150
MYB	F: ATGGCAGAAAGTACTAAACCC R: CAATTCTCCCCTTTAAGTGCTTG	137
UGT1A1	F: GGACTCGGCTCTGCTCTTAT R: GGAAAGGGTCTGTCAAAACG	107
PFKFB3	F: ACTGGAGCGGCAGGAGAACG R: GGGCTGGCTAGTGGGGTGAC	276
NDST4	F: CTGACCCTATTGTCCTCCTG R: ATTTCCCTTTGCCATTGTCT	156
CAPN6	F: AGGTATGGAACAAGCGAAAG R: AAATGAAAGGATGGAAGAGC	233
MSTRG.82376	F: CTTGCCTAAAGTGACATCGTT R: TGGCTCCAAAATAATTGCCTT	134
MSTRG.135103	F: AAGAGAACTTTACATTGGCTTG R: CCATAAAATAGTCTGCACGTT	133
MSTRG.202543	F: CGTAAGTCGAGAGCACCTGCC R: ACTTCTTGAGTTGCAGCAAGGGG	159
MSTRG.113677	F: TTTGGAAAACAAACCGACCAC R: TTATGATGCCTCCTGTTCAGC	159
MSTRG.123757	F: CCTATGCACAAAGTGTCACC R: CAGATCTTAGTTCCACGGTCA	122
MSTRG.201635	F: ACTGATGCGTCTCTAAACCC R: CCACAACTACTCATGCACGAGA	110
MSTRG.134746	F: GCACATCTTTAGGAAATTCGTT R: TATTAACACAACAAGCGAGGG	181
MSTRG.134749	F:TGCCTTCCAAATGTTTAGCTG R: TTTGGCCCTATTACATCCCAT	158
*β*-Actin	F:CCAACCGTGAGAAGATGACC R:CCCGAGGCGTACAGGGACAG	97

### Statistical analyses

All data were expressed as “means ± SD” At the time of comparison, a Student’s *t*-test was performed, and *P* < 0.05 was considered statistically significant.

## Results

### Identification and characterization of lncRNA

A total of 1,489,144,532 clean single-end reads were obtained by sequencing all 12 libraries. Each library single-end reads of obtained were above 11.9 million. Reads were then aligned onto the Ovis aries reference genome using HiSAT2. Approximately 90% to 94% of the reads were successfully aligned to the Ovis aries reference genome (Table 2).

**Table 2:** Summary of raw reads after quality control and mapping to the reference genome.

Sample	Raw Reads Number	Clean Reads Number	Clean Reads Rate(%)	Q30 (%)	Mapped Reads	Mapping Rate
bb_MF_U1	135,419,598	126,434,304	93.36	93.09	118,051,728	0.9337
bb_MF_U2	127,018,500	120,698,366	95.02	91.21	109,786,626	0.9096
bb_MF_U3	132,063,170	124,866,340	94.55	92.18	116,113,348	0.9299
bb_ML_U1	130,726,162	124,922,082	95.56	92.12	114,723,516	0.9184
bb_ML_U2	135,748,762	129,544,454	95.43	92.17	119,098,890	0.9194
bb_ML_U3	131,043,356	125,949,570	96.11	92.38	114,983,236	0.9129
bb_SF_U1	125,136,584	119,399,058	95.41	91.84	109,070,962	0.9135
bb_SF_U2	127,073,636	122,218,070	96.18	94.32	113,079,584	0.9252
bb_SF_U3	127,710,354	121,356,262	95.02	94.51	114,079,986	0.9400
bb_SL_U1	129,160,302	124,460,366	96.36	91.16	113,539,182	0.9123
bb_SL_U2	135,112,128	128,428,200	95.05	91.75	118,616,606	0.9236
bb_SL_U3	125,135,556	120,867,460	96.59	93.94	111,564,887	0.9230

**Figure 1:**
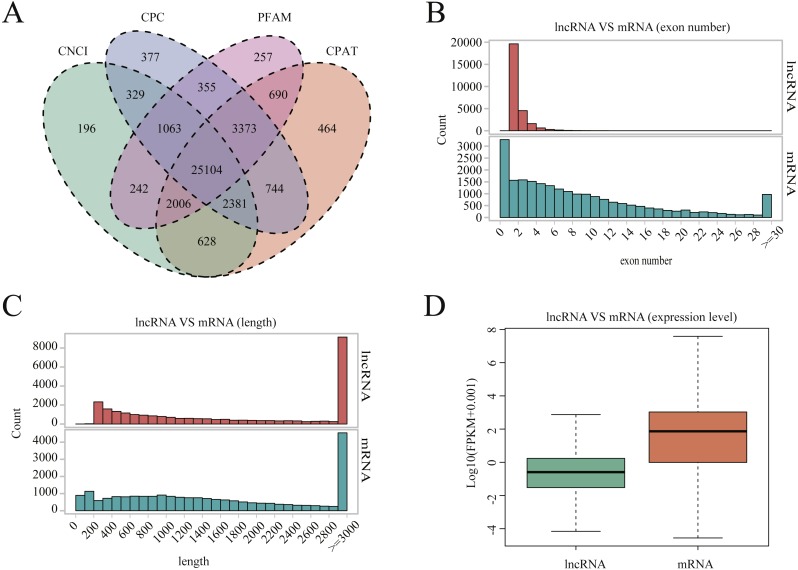
LncRNA characterization and gene expression. (A) Venn diagram for screening results of lncRNAs by four software (CNCI, CPC, CAPT and PFAM). The sum of the numbers in each large circle represents the total number of noncoding transcripts of the software, and the overlapping parts of the circle represent the noncoding transcripts common to the software. (B) The exon number distribution of lncRNA and mRNA. (C) The length distribution of lncRNA and mRNA. (D) The expression level of lncRNA and mRNA.

A total of 25,104 lncRNAs were identified in uterine tissues of the 12 ewes using four programs: CNCI, CPC, PFAM, and CAPT (Fig. 1A); 20,908 mRNAs and 16,016 novel transcripts were identified. Many lncRNAs have only two or three exons, whereas mRNAs contain a wide range of exons from two to thirty (Fig. 1B). Overall, the distribution of lncRNAs and protein-coding gene lengths were consistent, and the transcript lengths of lncRNAs were longer than those of mRNAs (Fig. 1C).

### Gene expression levels and differential expression analysis

**Figure 2:**
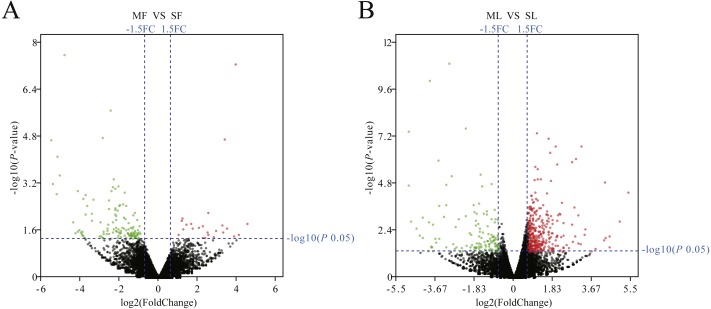
Analysis of differentially expressed genes. (A) Differentially expressed genes in the follicular phase. (B) Differentially expressed genes in the luteal phase. Red, green, and grayness dots in the graph represent transcripts that were significantly upregulated, downregulated and unchanged between polytocous and monotocous sheep respectively.

The Fig. 1D box plot shows that lncRNA transcript expression levels were all lower than those of mRNAs in the uterus of both polytocous and monotocous Small Tail Han ewes. Based on a fold change of >1.5 and a false discovery rate of <0.05, in the follicular phase, 242 lncRNA transcripts were up-regulated and 231 were down-regulated, and 33 mRNA transcripts were up-regulated and 133 were down-regulated in the PG ewes (Fig. 2A, Tables S1 and S2). Moreover, in the luteal phase, 330 lncRNA transcripts were up-regulated and 637 were down-regulated, and 359 mRNA transcripts were up-regulated and 146 were down-regulated in the PG ewes (Fig. 2B, Tables S3 and S4).

### GO annotation and KEGG enrichment analysis of differentially expressed genes

**Figure 3:**
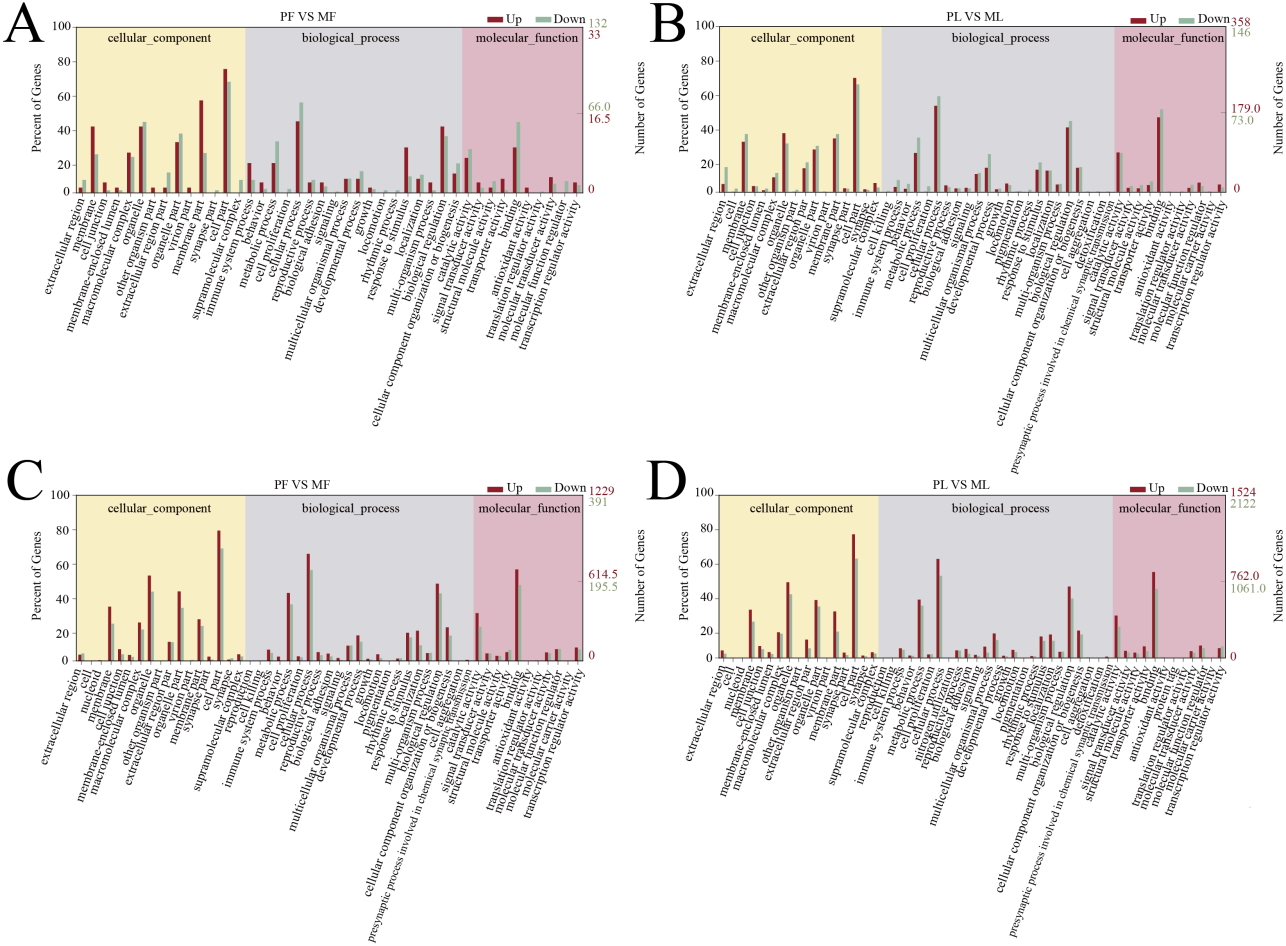
GO enrichment analysis of DE-mRNAs and target genes of DE-lncRNAs. (A) The DE-mRNAs GO enrichment analysis in the follicular phase. (B) The DE-mRNAs GO enrichment analysis in the luteal phase. (C) The DE-lncRNAs target genes GO enrichment analysis in the follicular phase. (D) The DE-lncRNAs target genes GO enrichment analysis in the luteal phase.

A total of 166 follicular phase and 505 luteal phase differentially expressed mRNAs were analyzed by GO analysis (*P* < 0.05); all were categorized into biological processes, cellular components, and molecular function (Tables S5 and S6). Reproductive process was enriched in the top 10 terms, which indicates that some genes regulate sheep reproductive traits in the follicular phase (Fig. 3A) and luteal phase (Fig. 3B) of polytocous and monotocous sheep.

**Figure 4:**
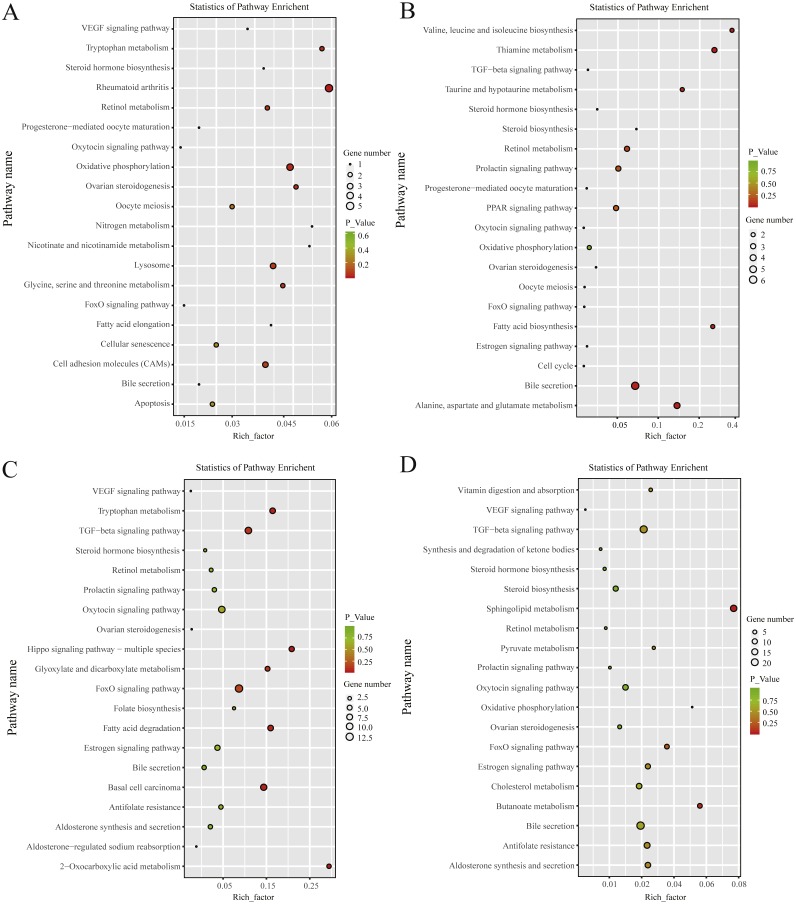
KEGG pathway analysis of differentially expressed mRNAs and lncRNAs. (A) Twenty KEGG enrichment pathways for differentially expressed mRNAs are presented in the follicular phase. (B) Twenty KEGG enrichment pathways for differentially expressed mRNAs are presented in the luteal phase. (C) Twenty KEGG enrichment pathways for differentially expressed lncRNAs are presented in the follicular phase. (D) 20 KEGG enrichment pathways for differentially expressed lncRNAs are presented in the luteal phase.The longitudinal and horizontal axis represents the enrichment pathways and Rich factor (amount of differentially expressed genes enriched in the pathway/amount of all genes in background gene set) of these pathways, respectively. Spot size represents the number of differentially expressed genes enriched in each pathway, and the color of the spot represents the *P*-value of each pathway.

KEGG pathway analysis revealed that, in the follicular phase, the differentially expressed mRNAs were assigned to 127 pathways (Table S7); they participate in ovarian steroidogenesis, steroid hormone biosynthesis, oxytocin signaling pathway, cell cycle, retinol metabolism and other critical regulatory processes (Fig. 4A). In the luteal phase, the differentially expressed mRNAs were distributed in 240 pathways (Table S8); they participate in the prolactin signaling pathway, steroid biosynthesis, steroid hormone biosynthesis, ovarian steroidogenesis, TGF-β signaling pathway, estrogen signaling pathway and other important regulatory processes (Fig. 4B). These results indicated that the differentially expressed genes in the ovine uterus of PG and MG ewes are involved in reproduction performance.

### LncRNA target genes and functional analysis

The genes transcribed within a 50-kb window upstream or downstream of the lncRNAs were considered cis-target genes, while targets in trans were predicted via calculating the expressed correlation (correlation coefficient ≥ 0.9) with lncRNAs.

In the follicular phase, three annotated lncRNAs corresponded to 11 target genes, and 470 novel lncRNAs corresponded to 1,578 target genes. GO terms of these lncRNA targets were enriched for several processes, such as reproductive process and cell proliferation (Fig. 3C, Table S9). KEGG analysis of these differentially expressed lncRNA target genes revealed that they were enriched in the foxo signaling pathway, TGF-β signaling pathway, estrogen signaling pathway, cell cycle, steroid hormone biosynthesis, ovarian steroidogenesis and retinol metabolism (Fig. 4C, Table S10).

In the luteal phase, 19 annotated lncRNAs corresponded to 157 target genes, and 948 novel lncRNAs corresponded to 3108 target genes. GO terms of these lncRNA targets were enriched for several processes, such as reproductive process, cell proliferation and metabolic process (Fig. 3D, Table S11). KEGG analysis of these differentially expressed target genes of lncRNAs revealed that they were enriched in the foxo signaling pathway, estrogen signaling pathway, TGF-β signaling pathway, ovarian steroidogenesis and VEGF signaling pathway (Fig. 4D, Table S12).

### LncRNA–mRNA co-expression network analysis

**Figure 5:**
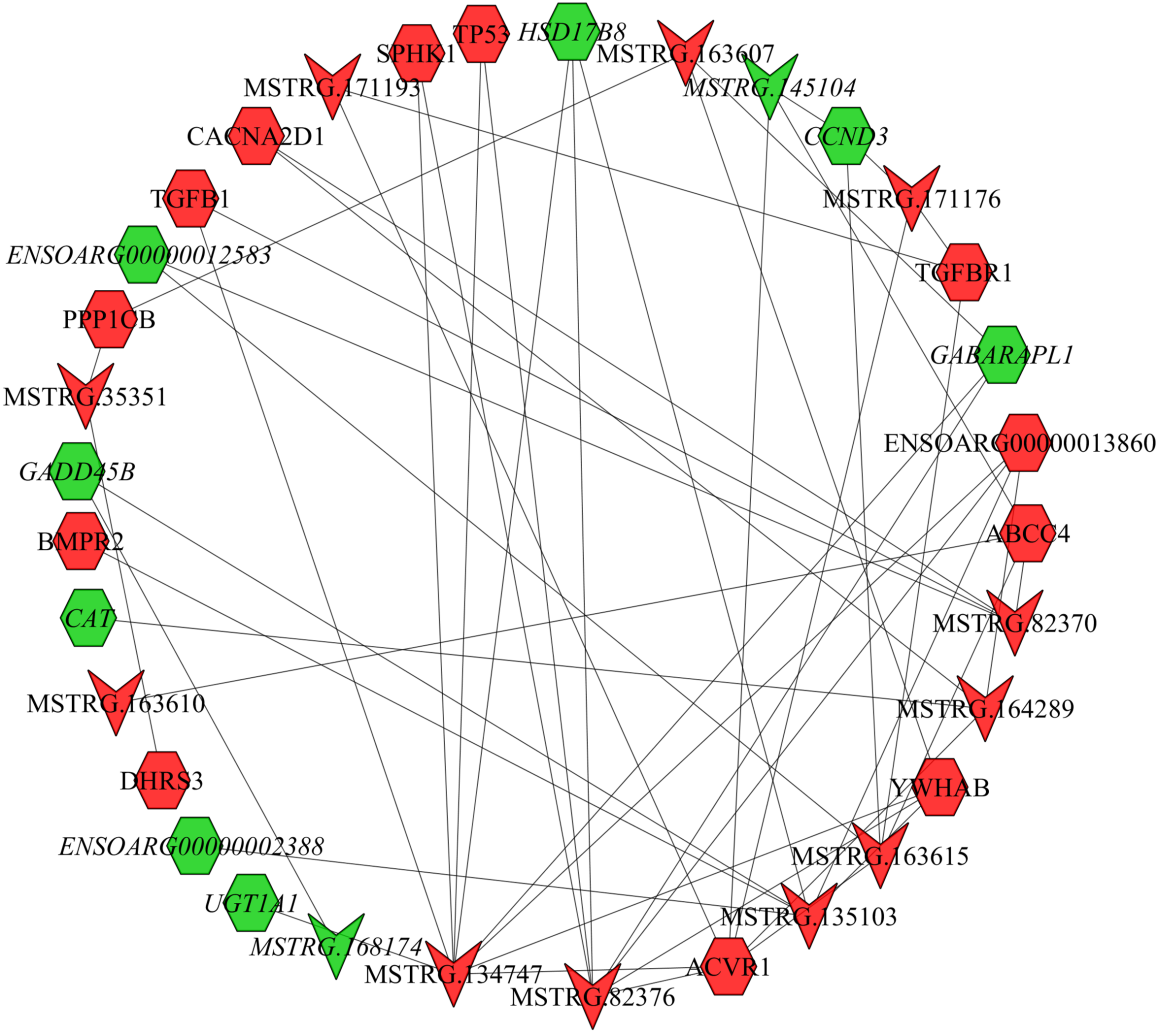
The network between DE-mRNAs and DE-lncRNAs in the follicular phase. Nodes represent lncRNA or mRNA, edges represent the interaction between lncRNAs and mRNAs. Red (regular) represents up-regulation and green (italic) represents down-regulation. Hexagons and inverted triangles represent mRNAs and lncRNAs, respectively.

**Figure 6:**
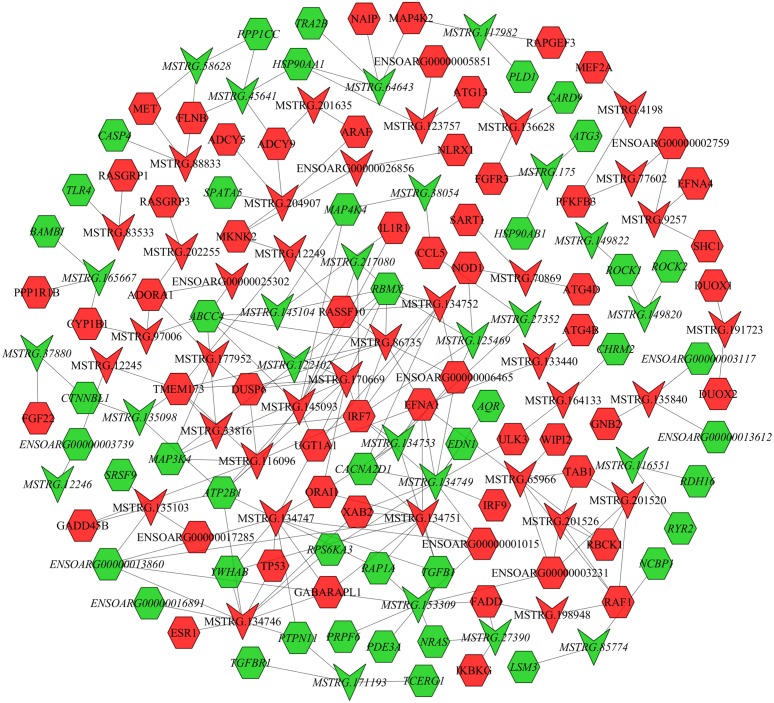
The network between DE-mRNAs and DE-lncRNAs in the luteal phase. Nodes represent lncRNA or mRNA, edges represent interaction between lncRNAs and mRNAs. Red (regular) represents up-regulation and green (italic) represents down-regulation. Hexagons and inverted triangles represent mRNAs and lncRNAs, respectively.

In the follicular phase, lncRNA-mRNA co-expression networks were constructed using 38 differentially expressed lncRNAs and 46 target genes involved in reproductive-related pathways. As shown in Fig. 5, some differentially expressed lncRNAs were at the center of the network, such as MSTRG.134747, MSTRG.82376, MSTRG.135103, MSTRG.82370, and MSTRG.163615 (Table S13). In the luteal phase, lncRNA-mRNA co-expression networks were constructed using 174 differentially expressed lncRNAs and 164 target genes involved in reproductive-related pathways. As shown in Fig. 6, some differentially expressed lncRNAs were at the central positions of the network, such as MSTRG.134747, MSTRG.134749, MSTRG.134746, MSTRG.134751, MSTRG.170669, MSTRG.153309, MSTRG.116096, MSTRG.134753, and MSTRG.135103 (Table S14). The network model showed that each lncRNA was co-expressed with polytocous genes, which indicates mutual regulation of lncRNA and mRNA in reproduction.

### RNA-Seq data validation by real-time PCR

**Figure 7:**
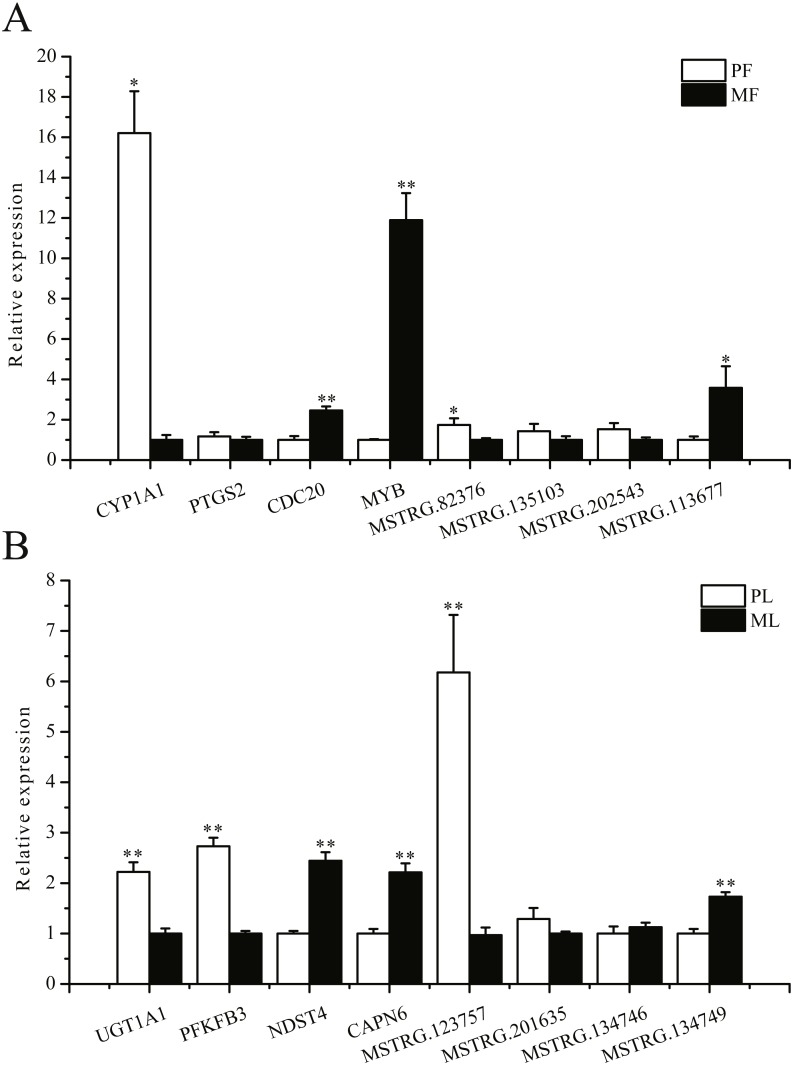
qRT-PCR verification of the differentially expressed genes. (A) The qRT-PCR verification of the differentially expressed genes in the follicular phase. (B) The qRT-PCR verification of the differentially expressed genes in the luteal phase. *: *P* < 0.05; **: *P* < 0.01.

To further validate the sequencing data, we selected eight involved in reproductive-related differentially expressed mRNAs and eight differentially expressed lncRNAs of targeted reproductive-related genes, and determined their expression levels by qRT-PCR (Fig. 7). The expression of each mRNA or lncRNA in the PG and MG ewes in the follicular and luteal phases (Figs. 7A, 7B) were consistent with those obtained by sequencing.

## Discussion

Reproduction capacity has important impacts on the profitability of sheep. More and more evidence indicates the important roles of lncRNAs in sheep reproduction (Feng et al., 2018). Some studies have also found that lncRNAs involve in Gonadgenesis (Mulvey et al., 2014), Sex hormone responses (Li et al., 2013), Sex determination (Hansen et al., 2013), Spermatogenesis (Arun et al., 2012) , Meiosis (Mau et al., 2013), and Placentation (Gao et al., 2012); (Keniry et al., 2012). Such as lncRNA *MSTRG.259847.2* regulates its target gene SMAD2 by cis, affects the interaction of SMAD2 with GDF9 and FSHB to regulate FSH synthesis (Zheng et al., 2019). It is well known that uterine functions such as immunity, energy supply and uterine receptivity, play a vital roles in reproductive process. However, current research on lncRNAs is mainly focused on the ovaries (Miao & Qin, 2015; Miao et al., 2016a; Miao et al., 2016b). In this study, RNA-Seq technology was used to analyze and compare the gene expression profiles of mRNAs and lncRNAs in the uteri of sheep in different phases of the estrous cycle. Further analysis of the interaction networks between mRNAs and lncRNAs revealed that these differentially expressed mRNAs and lncRNAs may play vital roles in sheep reproduction.

GO and KEGG enrichment analysis indicated that the differentially expressed genes are mainly involved in ovarian steroidogenesis, retinol metabolism, the oxytocin signaling pathway, steroid hormone biosynthesis, and the foxo signaling pathway. Analysis of differential lncRNA–mRNA co-expression patterns and functional analysis of target genes revealed that lncRNA affects sheep fecundity by modulating genes associated with the above signaling pathways and biological processes. In the follicular phase, these pathways were enriched with five differentially expressed genes (*CYP1A1*, *PTGS2*, *RDH12*, *CDC20*, *CCNA1*) and five lncRNA target genes (*MSTRG.134747*, *MSTRG.82376*, *MSTRG.135103*, *MSTRG.82370*, *MSTRG.163615*). In the luteal phase, these pathways were enriched with 12 differentially expressed genes (*RDH16*, *ALPL*, *ABCC1*, *SLC5A1*, *SCTR*, *LHB*, *CDKN1A*, *CYP24A1*, *BAMBI*, *UGT1A1*, *CREB3L4*, *GAD1*) and nine lncRNA target genes (*MSTRG.134747*, *MSTRG.134749*, *MSTRG.134746*, *MSTRG.134751*, *MSTRG.170669*, *MSTRG.153309*, *MSTRG.116096*, *MSTRG.134753*, *MSTRG.135103*).

*CYP1A1* both regulates estrogen activity in the ovary (Ptak, Ludewig & Gregoraszczuk, 2008) and catalyzes retinol metabolism (Wang et al., 2012). Knocking out *CYP1A1* could limit germ cell differentiation (Li et al., 2017). Prostaglandins are involved in regulation of many reproductive events, such as ovulation, corpus luteum regression, implantation, and pregnancy establishment (Karim & Hillier, 1979). *PTGS2* is a critical regulatory enzyme for prostaglandin biosynthesis, which enzymatically converts fatty acid precursors to prostaglandin G during prostaglandin biosynthesis (Wang & Dey, 2005). The possible role of *LHB* up-regulation is to increase the release of bioactive *LH* into the uterine environment during early pregnancy and exert paracrine effects that prepare the uterus for conceptus implantation (Pares et al., 2008). The litter size of the polytocous group was higher than that of monotocous group. In this study, the expression levels of *CYP1A1*, *PTGS2*, and *LHB* in the polytocous group were significantly higher than those in the monotocous group. This finding indicated that *CYP1A1*, *PTGS2*, and *LHB* might promote reproductive performance in the polytocous group. Moreover, *MSTRG.163615* and *MSTRG.82370* can trans-regulate *LHB*. *MSTRG.134747*, *MSTRG.134751*, *MSTRG.134753*, and *MSTRG.153309* can trans-regulate *TGFB1*, which is enriched in the ovarian steroidogenic pathway. *TGFB1* is abundantly expressed in the endometrium, and its proteins are secreted by endometrial cells and macrophages into uterine fluid (Goteri et al., 2015), and thus regulates uterine function. These results indicate that the genes enriched in the ovarian steroidogenic pathway mainly regulate endometrial and uterine endocrine function, which may affect embryo implantation.

Retinol metabolism and its active metabolites play a dual role in the reproductive tract (Ma et al., 2012). Retinoic acid (RA) is an active metabolite of retinol, and its metabolic site is the endometrial epithelium (Ozaki et al., 2017). RA is an essential morphogen during embryonic and fetal development. However, excessive retinoic acid inhibits embryo implantation (Geelen, 1979; Huang et al., 2005; Huang et al., 2003). *RDH12* and *RDH16* most efficiently produce retinal reductase, which affects embryo implantation by promoting retinol metabolism (Pares et al., 2008; Pavez et al., 2009). *UGT1A1* was expressed and regulated estrogen metabolism in the endometrium of uterine tissue (Duguay et al., 2004). In this study, *CYP1A1* and *UGT1A1* expression levels in the polytocous group were significantly higher than those of the monotocous group. *RDH12* and *RDH16* were significantly lower in the polytocous group compared with the monotocous group. This finding indicated that *CYP1A1* and *UGT1A1* might promote reproductive performance in polytocous sheep, and *RDH12* and *RDH16* might inhibit reproductive performance in polytocous sheep. *CYP1A1* and *UGT1A1* were up-regulated, whereas *RDH12* and *RDH16* were down-regulated in uterine tissue in the polytocous group. This result may be due to *CYP1A1* and *UGT1A1* catalyzing retinol metabolism to produce RA, whereas *RDH12* and *RDH16* catalyze RA metabolism, and RA plays an important role in embryo implantation and development. Moreover, *MSTRG.134747* might target *UGT1A1* in the retinol metabolic pathway to regulate intrauterine embryo implantation.

Oxytocin promotes uterine smooth muscle contraction and stimulates lactation during childbirth (Gimpl & Fahrenholz, 2001), and is also related to the fetal brain, heart, and kidney development and function (Ceanga, Spataru & Zagrean, 2010; Jankowski et al., 2004; Paquin et al., 2002). In this study, the differentially expressed genes *PTGS2* and *CDKN1A* were enriched in the oxytocin signaling pathway. *PTGS2* and *CDKN1A* were up-regulated in uterine tissue in the polytocous group, which indicates that *PTGS2* and *CDKN1A* may regulate sheep reproductive performance through the oxytocin signaling pathway. Additionally, *MSTRG.134747*, *MSTRG.135103*, and *MSTRG.82376* can trans-regulate *RHOA*; *MSTRG.134749* can trans-regulate *EDN1; MSTRG.201520, MSTRG.201526* and *MSTRG.65966* can trans-regulate *TAB1*; and *MSTRG.153309* can trans-regulate *FADD*, which is enriched in the oxytocin signaling pathway.

The interaction between development of conceptus and maternal endometrium is critical for establishing and maintaining pregnancy, and is regulated by many factors, including steroid hormones, prostaglandins, and cytokines (Bazer & Johnson, 2014). Vitamin D is a well-known secosteroid hormone involved in the regulation of cell proliferation and reproduction in mammals (Blomberg Jensen, 2014). *CYP24A1* is a metabolizing enzyme of vitamin D, which can promote vitamin D catabolism and affect sheep reproduction, is expressed in the endometrium in a pregnancy-specific manner as well as in the allantoic tissues of the villus during pregnancy (Jang et al., 2017; Liu & Hewison, 2012). *SLC5A1* is a sodium-dependent glucose transporter that moves glucose against its concentration gradient (Freking et al., 2007). Conceptus development depends on the energy provided by uterine secretions. During this process, the individual secretes E2, and the number of *SLC5A1* transporters in endometrial epithelial cells increases, thereby increasing the transport of glucose to the endometrium and providing nutrients for conceptus development (Steinhauser et al., 2017). In this study, *CYP24A1* was down-regulated, and *SLC5A1* was up-regulated in uterine tissue in the polytocous group, which indicates that *CYP24A1* might inhibit reproduction by promoting VD catabolism, and *SLC5A1* helps supply energy between the endometrium and embryo to promote reproductive performance.

## Conclusions

In summary, the uterus plays a vital role in sheep reproductive processes; for example, uterine gland secretion, uterine volume, and the endometrial immune system can affect sheep reproductive performance. These functions of the uterus are achieved through the regulation of different signaling pathways and related genes. In this study, we showed differential mRNA and lncRNA expression profiles associated with sheep prolificacy and constructed a network of interactions between lncRNAs and mRNAs. Additionally, we used the KEGG pathway to enrich the mRNAs and lncRNAs involved in sheep reproduction. Our study lays a solid foundation that may help elucidate the regulatory mechanisms of sheep mRNAs and lncRNAs.

##  Supplemental Information

10.7717/peerj.6938/supp-1 Supplemental Information 1 Raw data applied to the screening and functional analysis of differentially expressed genes in PG and MG in the follicular and luteal phases, respectively Table S1: Differentially expressed lncRNAs in the follicular phase
Table S2: Differentially expressed mRNAs in the follicular phase
Table S3: Differentially expressed lncRNAs in the luteal phase
Table S4: Differentially expressed mRNAs in the luteal phase
Table S5: The DE-mRNAs GO enrichment analysis in the follicular phase
Table S6: The DE-mRNAs GO enrichment analysis in the luteal phase
Table S7: KEGG enrichment analysis of mRNAs in the follicular phase
Table S8: KEGG enrichment analysis of mRNAs in the luteal phase
Table S9: The DE-lncRNAs target genes GO enrichment analysis in the follicular phase
Table S10: KEGG enrichment analysis of lncRNAs in the follicular phase
Table S11: The DE-lncRNAs target genes GO enrichment analysis in the luteal phase
Table S12: KEGG enrichment analysis of lncRNAs in the luteal phase
Table S13: The network between DE-mRNAs and DE-lncRNAs in the follicular phase
Table S14: The network between DE-mRNAs and DE-lncRNAs in the luteal phase.

## Additional Information and Declarations

### Data Availability

The following information was supplied regarding data availability: Data is available at the Sequence Read Archive (SRA): SRP173986
